# Interaction between microorganisms and dental material surfaces: general concepts and research progress

**DOI:** 10.1080/20002297.2023.2196897

**Published:** 2023-04-05

**Authors:** Yan Tu, Huaying Ren, Yiwen He, Jiaqi Ying, Yadong Chen

**Affiliations:** a Department of Endodontics, Stomatology Hospital, School of Stomatology, Zhejiang University School of Medicine, Hangzhou, China; b Zhejiang Provincial Clinical Research Center for Oral Diseases, Key Laboratory of Oral Biomedical Research of Zhejiang Province, Cancer Center of Zhejiang University, Hangzhou, China; c School of Stomatology, Zhejiang University School of Medicine, Hangzhou, China

**Keywords:** Bacterial adhesion, dental materials, surface properties, biological contamination, intelligent dental material surfaces

## Abstract

Bacterial adhesion to dental materials’ surfaces is the initial cause of dental materials-related infections. Therefore, inhibiting bacterial adhesion is a critical step in preventing and controlling these infections. To this end, it is important to know how the properties of dental materials affect the interactions between microorganisms and material surfaces to produce materials without biological contamination. This manuscript reviews the mechanism of bacterial adhesion to dental materials, the relationships between their surface properties and bacterial adhesion, and the impact of bacterial adhesion on their surface properties. In addition, this paper summarizes how these surface properties impact oral biofilm formation and proposes designing intelligent dental material surfaces that can reduce biological contamination.

## Introduction

Bacteria exist in almost every corner of the Earth. They can be found from deep-sea niches to the subsurface, and even in the human digestive tract. Among all the bacteria, some harmful bacteria could cause some human health issues, such as chronic infectious diseases [1]. Therefore, understanding the mechanisms of bacterial adhesion and biofilm formation [1,2] is essential in bacterial pathogenicity research and biofilm-related infection control.

The oral microbiome is one of the most diverse microbial communities in humans, with an estimated 600 species at present (10^9^ cells/mL [3] were found in human salivary samples). Dental diseases such as dental caries and periodontitis, which are closely connected to the oral microbiome, have imposed a significant burden on human health. Furthermore, the oral microbiome is associated with health issues outside the oral cavity. Oral epithelial cells shed three times a day, and the rapid renewal and turnover of oral epithelial cells is a very effective way to reduce bacterial adhesion. In contrast, biofilms are more likely to form on rarely renewed surfaces, such as teeth, dental restorations, dental prostheses, and dental implants, leading to severe oral health problems like dental caries, periodontitis, and implant failure [4]. In the oral environment, saliva, with glycoproteins (mucin), phosphoproteins, α-amylase, and some other molecular structures, such as glucosyltransferase, often adheres to the surfaces of teeth, dental restorative materials, dental prostheses, and dental implants, forming acquired pellicle [5]. During the formation of the acquired pellicle, salivary proteins or glycoproteins are initially adsorbed to the tooth surface to form a non-structured, cell-free film. This film is quickly formed on freshly cleaned tooth surfaces within minutes, rapidly thickens to several layers in 1‒2 hours, with a thickness of 1‒20 μm, and is composed of proteins, carbohydrates, and fats. The formation of the acquired pellicle can promote the adhesion, colonization, and coaggregation of bacteria at an early stage. However, different dental materials exhibit different acquired pellicles on the surface, in the formation of the acquired pellicle, and in the structure of the acquired pellicle [6]. Therefore, further research is required on the influence and mechanism of properties of different dental materials on the formation of the acquired pellicle.

**Figure 1.**
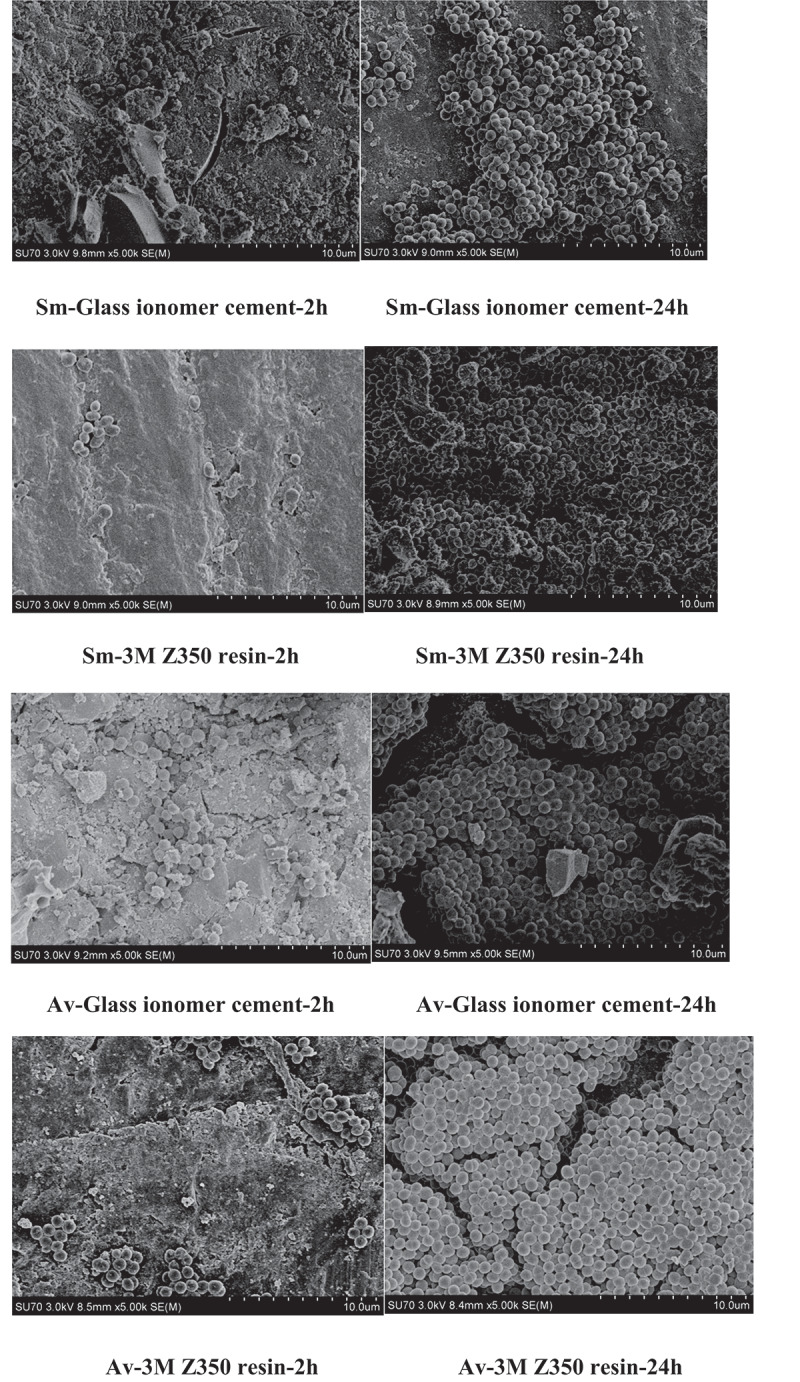
Electron microscopy pictures of biofilms on different dental materials.

In the complex oral microbiome, oral *Streptococcus* species, including *Streptococcus mutans* and *Streptococcus sanguis*, can colonize the surface of the acquired pellicle [7] at an early stage through adhesin and electric charge interactions. As the plaque matures, the number of bacterial species (such as *Porphyromonas gingivalis*, *Fusobacterium nucleatum*, and *Actinomycetes aggregate)* gradually increases [8,9]. Coupled with various hosts and dietary factors, bacteria are connected through adhesion and coaggregation. Then the colonized bacteria rapidly divide, multiply, and grow, increasing the numbers and diversity of bacteria and finally forming complex oral biofilms [10]. Glucosyltransferase in the acquired pellicle can produce extracellular polysaccharides from sucrose, increasing the affinity of bacteria and promoting the development of cariogenic biofilms [11]. Therefore, a high-sugar diet can significantly affect the dynamic adhesion of bacteria and promote the formation of biofilms. In the oral environment, biofilms can be found on the surface of various dental materials, including prosthetic, endodontic, orthodontic, and implant materials [12]. In our previous research, we demonstrated the formation of biofilms on the surfaces of dental restorative materials and sealers in root canal treatment in vitro. In that study, some dental materials were coated with sterilized saliva and incubated at 37ºC for 30 min. Then the materials were immersed in the corresponding supragingival bacterial solution and cultured under anaerobic conditions at 37ºC for 2‒24 h. The adhesion of bacteria was observed under a scanning electron microscope [13] (Figure1). We also asked some volunteers to wear a plaque-collection device equipped with four restorative material sheets for two days, collected the plaque samples, and applied Miseq sequencing to obtain template DNA fragments for microbial diversity analysis. The results showed that the types and abundance of bacterial species were significantly influenced by the physicochemical properties of restorative materials [14] (Table 1). In addition, other factors, such as diabetes, smoking, AIDS, etc., can change the local host environment and promote biofilm formation, leading to various oral diseases [15].

In the study, the materials were coated with sterilized saliva and incubated at 37°C for 30 min.Then the materials were immersed in the corresponding supragingival bacterial solution and cultured under anaerobic conditions at 37°C for 2-24 h. The adhesion of bacteria was observed under a scanning electron microscope. As time moved forward, bacteria connected with each other, reproduced and grew through adhesion copolymerization, and the number of bacteria in plaque increased, forming different kinds of complex bacterial biofilms.(*Sm*: *Streptococcus mutans*; *Av*: *Actinomyces viscosus*; Glass ionomer cement: dental restorative materials; 3 M Z350 resin, dental restorative materials.)

**Table 1.** Abundance of bacteria adhered to four materials at the generic level.

Bacterial abundance	ICON permeable resin	3 M Z350 composite resin	3 M glass ionomer	zinc phosphate cement
The highest abundance	Neisseria Porphyromonas Fusobacterium Rothia Granulicatella	Lautropia Granulicatella	Haemophilus Veillonella Prevotrlla	Streptococcus Gemella Acinetobacter
The lowest abundance	Veillonella Acinetobacter	Haemophilus	Streptococcus Gemella Lautropia Granulicatella	Neisseria Porphyromonas Fusobacterium Rothia Granulicatella

These bacteria with the above differences play an important role in oral diseases. They are gram-positive/negative bacteria. These bacteria are closely related to caries, periodontal diseases and gingival diseases. The differences in the abundance of adhesive bacteria among the four groups of materials are of clinical significance for the selection of restorative materials and the reduction of secondary caries after filling.

In dental treatments, microbial infection is the main cause of secondary caries, loss of restorations, and tooth pain, which is a concern for most dentists. Therefore, much research has been focused on reducing the number of bacteria in the restorative material and decreasing the incidence of dental pain after restorative procedures.

There are many ways by which bacteria can approach the surface of dental materials, including Brownian motion, fluid movement, deposition, and interaction with other cells [16]. As the interaction between bacteria is very complex, no theoretical model can accurately describe the process of bacterial adhesion to different dental materials. Therefore, it is imperative to research the interactions of bacteria and dental materials, which is the key to controlling biofilm formation.

## Mechanism of bacterial adhesion to dental materials

### Biofilm formation is a complex process starting from the bacterial adhesion to the surfaces of teeth, which can be divided into four stages in the oral cavity [17] (Figure 2)

1. Acquired pellicle formation: All the
  surfaces exposed to the oral environment are stably covered with a thin film composed of organic and inorganic molecules adsorbed to teeth, resin composites, and dentures. These molecules mainly originate from saliva and serve as bacterial receptors.
2. Reversible adhesion: Brownian motion and salivary flow can lead to the initial transport of individual microbial cells or microbial aggregates to the nearby solid matrix. This stage involves weak, long-distance, and reversible physicochemical interactions.
3. Irreversible adhesion: This stage includes the formation of a strong anchoring force by specific interactions between bacteria and the surface, such as covalent, ionic, or hydrogen bonds.
4. Coadhesion and mature biofilm formation: In the last stage, secondary colonizing bacteria adhere to the receptors of attached microorganisms, resulting in biofilm development and proliferation.

**Figure 2.**
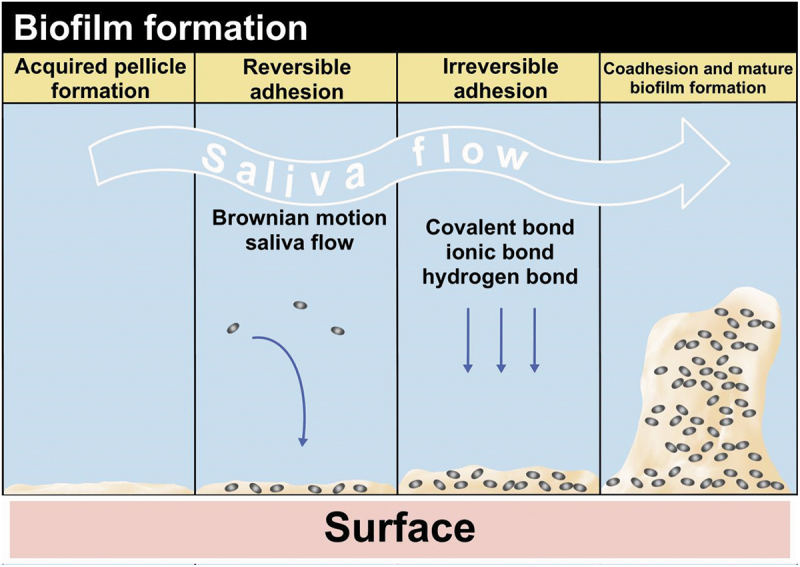
Mechanism of bacterial adhesion to dental materials.

The biofilm formation can be divided into four stages in the oral cavity : acquired pellicle formation, reversible adhesion, Irreversible adhesion, co-adhesion and mature biofilm formation.

Nowadays, with developments in science and technology, the model of the biofilm formation described above gradually shows limitations. Karin Sauer et al. [18] reported that although the previous model of biofilm formation was easy to understand, this model did not reflect the relevant microenvironments that develop within these biofilms. They depicted three major steps of biofilm growth: aggregation, growth, and disaggregation. We have noticed that the new model does not consider the surface a necessary condition for biofilm formation. In the oral environment, bacteria are mainly distributed on the enamel and dental material surfaces [19]. Some biofilms may exist in flowing saliva without a surface [18]. However, in the oral environment, because the biofilm in flowing saliva is difficult to study, researchers will pay more attention to the relationship between dental materials and microorganisms. Therefore, in this review, we will focus on the surface of dental materials.

### Dental materials and biofilm formation

The performance of oral surfaces affects the surface properties of the pellicle, initial bacterial adhesion, and biofilm formation. In particular, it significantly influences some dental materials, such as the restorations used to restore the missing dentin and/or enamel tissues, with a high risk of secondary caries after contacting the remaining tooth structure. In fact, clinical research has shown that secondary caries is still the main cause of restoration failure, which is intimately associated with biofilm formation on dental restorative materials [20,21].

#### Resin composites

Resin composites have become the most common oral restorative materials [21] because of their widespread application in anterior and posterior teeth, unique aesthetic characteristics, and the advantage of easy handling. These materials are composed of a polymer matrix, reinforcing fillers, a silane coupling agent, and some chemical components that promote the regulation of the polymerization process. The main monomer in the composite resin is Bisphenol A-glycidyl methacrylate (bis-GMA), which is usually mixed with other dimethacrylates. In addition, the new monomer has a high molecular weight and can reduce the composite resin shrinkage. Resin composites are classified according to their fillers, especially concerning their size. The latest development in resin composites is the introduction of nanoparticle composite fillers, which can improve the mechanical and aesthetic properties of resin composites by reducing the particle size and bringing about even distribution [22]. Developing restorative materials that are not conducive to microbial adhesion and colonization is the goal of contemporary dental materials science. The development of new resin composites and adhesive systems prolongs the release of antibacterial agents [23–27]; however, biofilm formation also depends on the surface characteristics of these materials [28,29]. Therefore, fine-tuning the physical and chemical properties of resin composites can prevent biofilm formation, laying a solid foundation for developing new resins.

#### Amalgam and metal bionic materials

Amalgam is a special type of alloy formed by mercury and one or more metals. The amalgam used for dental restorations has a long history. In 1896, G. V. Black of the United States carried out much research on improving the composition, properties, blending, and filling methods of silver amalgam, gradually making silver amalgam an ideal filling material. Orstavik et al. tested nine commercial dental amalgams for antibacterial properties in vitro and found that all displayed certain antibacterial properties [30]. The reason was that amalgam could release metal ions such as Ag, Cu, Sn, and Hg; therefore, it had certain antibacterial properties. Farrugia et al. found that amalgams had higher antimicrobial activity than adhesive materials [31]. However, now because of the toxic effect of mercury on the human body and its pollution potential, the rate of amalgam use in dentistry has decreased significantly. Combining the rigidity and antibacterial properties of metals to reduce toxicity has become the focus of scientific research. Silver-based biomaterials (AgBMs) have good antimicrobial properties, including penetrating microbial cell membranes, damaging genetic material, and causing bacterial protein and enzyme dysfunction. Research has shown that AgBMs are antibacterial materials with high efficiency and low toxicity [26].

#### Ceramic and ceramic nanomaterials

Since ceramic materials were introduced to the dental restoration field, they have been widely used because of their good biocompatibility and aesthetic effects. However, ceramics’ inherent defects, such as insufficient strength and brittleness, have significantly limited their application in dental restorations. Ceramic nanomaterials are composite materials. Nano-sized ceramic particles, whiskers, fibers, etc., are added to the ceramic matrix to improve the performance of ceramics. Ceramic nanomaterials can be synthesized by rapid cooling after heating at high temperatures, a nanoscale inorganic metalloid solid. Since ceramic nanoparticles are more stable than metallic nanoparticles and have lower production costs, they are widely used in dental restorative materials [32]. Imran et al. showed that hydroxyapatite-based nanoparticles could promote mineralization in early carious lesions by combining with the porous tooth structure caused by the caries process to increase its mineral content and hardness [33]. Another study showed that bioactive glass-based nanoparticles could improve the regeneration of dentin, cementum, and bone in pulp or periodontal tissues by adding variable doping materials to promote hard tissue regeneration [34]. Antimicrobial nanoparticle-conjugated ceramic minerals can provide dual functions to prevent the formation and development of dental caries.

## Relationship between dental material surface properties and bacterial adhesion

### Surface energy and hydrophobicity of dental material surfaces

Bacterial adhesion can be promoted or prevented by adjusting the hydrophobicity of dental materials. Therefore, hydrophobicity plays an important role in oral bacterial adhesion [7,35,36]. The surfaces of supragingival and subgingival biofilms are different in the oral environment. Regarding the supragingival environment, the biofilms formed on hydrophobic surfaces are fewer than those on hydrophilic surfaces. In contrast, there is no difference between hydrophobic and hydrophilic surfaces in the subgingival environment. This difference is attributed to the flow renewal rate on the gingiva and a higher rate of bacterial shedding from the hydrophobic surfaces due to the action of flow shear force [37].

In addition to the hydrophobicity of dental materials, the hydrophobicity of bacterial cells is also critical. Bacterial species have different surface hydrophobicity, affecting the adhesion between the two. The surface hydrophobicity of bacterial cells depends on the structure and composition of the relevant cell membranes, which is a primary performance of bacterial adhesion rate. Nonpolar molecules are responsible for hydrophobic behavior, such as lipoteichoic acids, teichoic acids, and S-layer on cell surfaces [38]. For example, it has been reported that hydrophobic *Streptococcus sanguis* can more tenaciously adhere to the surface of saliva-coated secondary pure cp-Ti alloy compared with hydrophilic *S. constellatus* [39].

**Figure 3.**
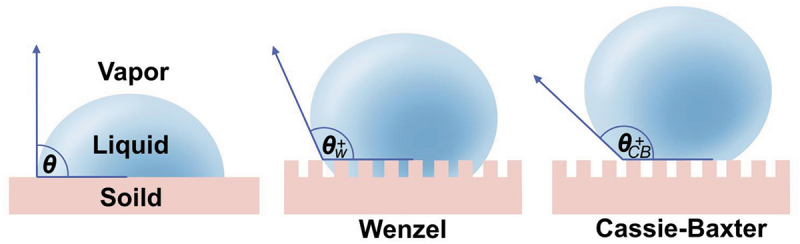
The Cassie-Baxter model.

In addition to some conventional hydrophobic surfaces, it has been reported that superhydrophilic surfaces can inhibit biofilm formation. In nature, many animals and plants have superhydrophobic surfaces, such as roses and lotus leaves, with a rolling angle of<10°. Because of their low surface energy and special roughness, they have a remarkable self-cleaning ability, and superhydrophobicity is the major factor [40]. Studies on these animals and plants in nature provide a deeper understanding of superhydrophobic surfaces. This is of great significance for the preparation of biomimetic materials. For example, the Cassie-Baxter liquid usually shows lower sliding and contact angle hysteresis than the Wenzel liquid. Based on a Cassie-Baxter model (Figure 3), the system achieved superhydrophobicity when the surface roughness was within an acceptable range [41]. Some durable superhydrophobic-layered biomimetic materials have been developed according to this theory [40].

The liquid droplets on the solid surface surrounded by gas form a contact angle: θ. If the liquid directly contacts the concave and convex surface of the solid surface microstructure, the liquid drop is in Wenzel state; If the liquid is only in contact with the convex surface of the microstructure, the liquid drop is in the Cassie-Baxter state. Cassie-Baxter liquid usually shows lower sliding angle and contact angle hysteresis than Wenzel liquid. According to the Cassie-Baxter model, when the surface roughness is within a proper range, the system reaches the Cassie state, thus achieving superhydrophobicity

Superhydrophilic surfaces can form water molecule surfaces with good antifouling performance. Since the strong interaction force between the surface and water makes the contact angle equal to or close to 0, this property weakens the interaction of matrix material, reducing cell adhesion. The features of Zwitterion-based mixed matrix membranes include enhanced water flux, fouling mitigation, and selectivity. They have emerged for oilsands process-affected wastewater as an improved membrane treatment. Zwitterionic structures with the advantage of superhydrophilicity have attracted significant attention in this field [42]. MPC (2-methacryloyloxyethyl phosphorylcholine) is well-known as a zwitterionic polymer/monomer exhibiting superhydrophilic properties [43]. However, the application of superhydrophilic surfaces in the oral cavity should still be studied. Interestingly, Venault et al. [44] reported that coating the surface with zwitterionic polymer SBMA could inhibit the adhesion of lysozyme because zwitterionic molecules could capture water to become superhydrophilic. Some relevant tests result reflected that 70% of oral bacteria adhering to human teeth were inhibited [44]. With the continuous advances in material science and manufacturing technology, there will be more interesting developments in this area in the future.

### Surface charge of dental materials

Surface charge plays a very significant role in the link between bacteria and dental material surfaces. Most bacterial cells are negatively charged. Therefore, bacterial adhesion to the surface of positively charged materials is easier, while the surface of negatively charged materials can resist bacterial adhesion. At the same time, if there are some cationic groups on the surface, such as quaternary ammonium salts and polyethyleneimine with antibacterial activity, the bacteria attached to the surface can be killed [45]. Theoretically, the bacterial adhesion controlled by surface charge will not work in the static system because the dead cells can reduce the surface charge, promoting the adhesion of other bacterial cells. It has been demonstrated that the flow shear force can stably remove the dead cells on dental materials, effectively reducing biofilm formation.

The surface charge can also influence the structure of biofilms for an extended period. Terada et al. used the radiation-induced graft polymerization of monomers to modify polyethylene sheets. They introduced diethylamine and sodium sulfite as corresponding functional groups in the experimental research process, opened the epoxy group of GMA under the action of a certain electrostatic force, and then analyzed these modified surfaces to determine their impact on the initial adhesion of *E. coli*. Statistical analysis of the experimental data showed that the main factor affecting the robustness of the *E. coli* biofilm was the surface charge property of the substrate, and the correlation with other factors was not obvious. Their research showed that the bottom layer surface based on RIGP had a strong regulatory role in the formation process of this film [46]. Other environmental factors in the complex system may complicate the surface charge impact on biofilms. For example, the formation of salivary pellicles induced a negative charge on the surface, mediating the adhesion of Ca^2+^ and promoting the adhesion of *Fusobacterium nucleatum* to the titanium surface [47]. Moreover, the performance of dental materials will significantly impact the density, configuration, and strength-related factors of the formed biofilm [16]. Therefore, further research is needed to clarify how surface charges affect the properties of the biofilm and determine whether surface charges can be customized to promote the integration of host cells and reject or kill bacterial pathogens that cause oral diseases.

### Surface roughness of dental materials

Among all the surface properties, surface roughness has become the main topic in the research on dental biofilms. The higher surface roughness generally promotes bacterial adhesion because the contact area increases [48], while the shear force decreases [37]. Therefore, a smooth surface can reduce biofilm formation [49]. It has been reported that a roughness of 0.2 µm is the maximum threshold to reduce bacterial adhesion on adjacent surfaces [50]. However, the exact influence of surface roughness on bacterial adhesion and biofilm formation is affected by bacterial cell size and other factors. As a result, no optimum roughness can inhibit the adhesion process [51]. Xing et al. [52] reported that microorganisms’ adhesion to the abutment of TiZr dental implants was positively correlated to the nano-roughness of its surface (<214 nm). In contrast, increasing the roughness of the ceramic surface from 0.2 μm to 2 μm was not conducive to biofilm formation [53]. These differences might be attributed to differences in material composition, roughness level, bacterial strain, and culture environment. All the surfaces in the oral cavity are covered by salivary pellicles [54], which can also change the nano-morphology [5], significantly affecting the surface roughness. In addition, in the presence of sucrose, bacterial enzymes can produce exopolysaccharides in situ, and the locally formed dextran can change the local environment and provide specific binding sites for some toxic species [11]. Therefore, it can be inferred that, to control biofilm formation, the material’s roughness should be protected against the influence of saliva and the impact of the adhesion performance of bacteria by changing the type of saliva/microproteins adsorbed by the material.

In addition to adhesion, we found that some surface topographical features can affect adherent cells’ stability. For example, bacterial cells were often penetrated by the nano-pillar arrays on the wing surface when incubated on cicada wings, resulting in bacterial cell death [55]. It is speculated that these activities are due to the stretching of bacterial cell membranes suspended among the nano-pillars. Therefore, the cicada wings were effective antibacterial agents, as opposed to anti-biofouling surfaces. Therefore, if the stretching degree is enough, it may cause cell membrane damage or even the death of adhesion cells [56]. Zinc oxide nanorods also impacted the bactericidal activity of *Staphylococcus epidermidis*. For example, when 15% *Staphylococcus epidermidis* was attached to zinc oxide nanorods, these nanoscale structures could kill these bacteria [57].

### Patterns on the surface of dental materials

**Figure 4.**
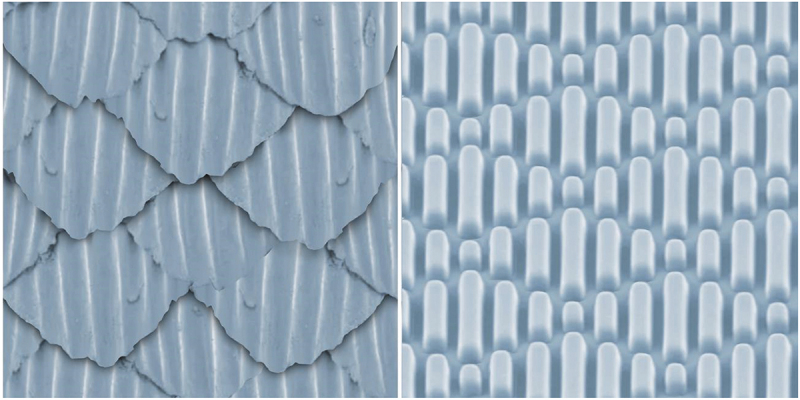
Sharklet surface.

Studies have consistently shown that the distribution of peaks and valleys on the surface of polymerized dental materials is crucial for microbial film formation [58,59]. The latest research has made significant progress regarding materials and their surfaces in controlling surface roughness and defining the surface topography pattern to control biofilm formation [60,61]. As a well-known example of a Sharklet surface (Figure 4), a shark’s skin is always smooth and clean because it is composed of millions of tiny tooth-like dermal denticles with a regular arrangement that forms a micron-scale structure, making it difficult for the adhesion process [62]. Compared with the flat surface of the same material, many micron and nanometer topographic patterns with different sizes and shapes can inhibit biofilm formation, including concave-convex squares, round and parallel channels of polydimethylsiloxane (PDMS) [63], cone patterns of silicone resin [64], ridge patterns on polydimethylsiloxane [65], strain-induced wrinkles on polydimethylsiloxane [66], and round poles on polystyrene [67]. Some well-defined nanostructures induce superhydrophobicity and reduce biological contamination [68]. In addition to pollution control, these surfaces can help clarify the interactions between bacteria and surfaces. Take the 10-µm-high square patterns on polydimethylsiloxane as an example. The associated conjugation of polydimethylsiloxane (PDMS) with a 10-µm raised pattern on the surface was studied using fluorescent-labeled *E. coli* donor and recipient strains. It can be inferred that, compared to smooth control, the square terrain pattern with side lengths of 20, 50, and 100 µm and a mode spacing of 10 µm or more promoted the formation and joining of biofilms. The vertical sides were the‘hot spots’ for bacterial adhesion compared to the patterns [69].

The figure on the left is a sketch of shark skin, while the figure on the right is the schematic diagram of Sharklet surface. Shark’s skin is composed of millions of tiny teeth like dermal denticles with regular arrangement, which form the micron equipment that make it difficult for bacteria to adhere to, so as the shark surface.

### Surface stiffness of dental materials

Of all the material properties, material stiffness has the least effect on the surface; therefore, there is limited literature on the stiffness of materials. Hervé et al. [70] studied bacterial adhesion in PDMS samples. They demonstrated that intrinsic performance related to PDMS substrates of varying hardness strongly affects bacterial adhesion, contrasting reported theories about bacterial active mechanical sensing. This theory provides new ideas for surface design. Sandra et al. [71] suggested that material stiffness could affect microbial adhesion, demonstrating that micrometer- and submicron-scale topographic patterns give surfaces unique properties independent of stiffness. Their experimental research results can provide support for clarifying the correlation between material hardness and bacterial physiology and lay a good foundation for proposing more bacterial adhesion inhibition technology. We also found that *Lactobacillus hungrier* [72] grew faster on the polyelectrolyte multilayer film with lower rigidity (Young’s modulus of 30 KPa) than on the film with higher rigidity (Young’s modulus of 150 KPa). Guegan [73] believed that the elastic modulus of agarose hydrogels could affect the formation of cell clusters of proteins adhering to bacterial cells. Yanrui et al. [74] reported that the hardness of polydimethylsiloxane (PDMS) affected the formation of *E. coli* biofilms. They found that shorter *E. coli* cells were easier to engulf than longer cells isolated from the surface of soft PDMS. In addition, macrophages were more active on hard PDMS surfaces and more efficient at phagocytosing *E. coli* cells. Song et al. speculated that the mechanism of material stiffness affecting the formation of bacterial biofilm was through influencing adhesion and cell movement. However, it is still unclear how stiffness is translated into changes in cell adhesion and biofilm growth [75]. These findings are beneficial for biomaterial designing to reduce infections. At this moment, we know that material stiffness can affect the adhesion and physiological activity of the adhering bacterial cells. Understanding these functions is very important for designing the surfaces of dental materials without biological contamination, especially the surfaces of new restorations, orthodontic materials, or implants.

## Effects of bacterial adhesion and biofilm formation on surface properties of materials

Bacteria have complex systems to sense environmental conditions. For example, in a study on *E. coli* adhesion to a 2.7-µm-high hexagonal topographic surface, *E. coli* could use flagella to adhere to the surface [76]. However, limited research is available regarding the detection of a surface mechanism basis, except for some induction research on surface humidity [77] and surface contact [78]. Cyclic GMP II is closely related to bacterial biofilm formation and can promote bacterial biofilm formation. Relevant studies found that in *Pseudomonas aeruginosa*, FleQ regulates the expression of the flagellum gene and extracellular polysaccharides and has a strong regulatory role in plant stress resistance. In the plant-friendly bacterium KT2440, the mutation of fleQ significantly decreased biofilm colonization, but the mechanism is not very clear. Studies have shown that the FleQ of *P. putida* interacts with a direct bond to the flagella and the promoter region of the EPS gene [79]. Another kind of surface sensing, called the Wsp regulation circuit, was found in recent research on *Pseudomonas aeruginosa*. In this system, WspA, which is homologous to membrane-bound chemoreceptors, senses the unknown surface signals (presumably related to mechanical pressure), leading to the increased production of c-di-GMP and finally causing biofilm formation [80]. Since these research studies were all based on the inductive contact of surfaces, the reaction mechanism of bacteria to the properties of certain materials remains to be studied. Research should also clarify bacterial induction of the surface.

In vitro research has shown that the adhesion and aggregation rate of bacteria on composite resin surfaces is higher than that on natural tooth surfaces [81–86] and confirmed by some in vivo studies. The surface characteristics of resin composites can affect biofilm formation. In addition, it was discovered that surface energy could be destroyed by the biological environment of the oral cavity [87,88]. The main energy of material degradation comes from the consumption of various enzymes, beverages, and foods [89], and the most important one is the role of cariogenic biofilms [90]. However, at present, our understanding of the impact of carcinogenic biofilm on the surface properties and microstructure of restorations is still limited.

The effect of the bacterial complex on surface topography has been thoroughly analyzed in vitro. For example, it was shown that this kind of biofilm growing on the surface for one month increased the roughness of the material surface but did not change its microhardness [91]. Moreover, this research result emphasized that increasing the surface roughness of composite resin could promote biofilm formation, and after biofilm formation, the roughness of composite resin would increase, forming a ‘vicious circle’ and reducing the longevity of the restoration. Another similar experimental result [92] regarding different repair materials showed that the biofilm on the surface of the experimental sample was material-dependent. In fact, the surfaces of materials exposed to *Streptococcus mutans* biofilm for one month exhibited micromorphological damage, increased surface roughness, and increased fluoride release from modified resin and conventional glass ions. Therefore, the deterioration of the surface properties of glass ions accelerated the biodegradation process of materials in the cavity. However, since in vitro research cannot completely reproduce the complex biodegradation process, further in vivo research is necessary. It suggests that good experimental design can imitate what happens in the oral cavity, providing clinical data [93].

Barbosa RP et al. used a self-controlled, double-blind, cross-sectional experiment to analyze the role of biofilm development and its cariogenic effect on surface microhardness. Polymer materials are more susceptible to caries than composite resin materials because the surface of the polymer is more easily damaged, and the surface hardness is lost. However, the results reflect that the surface hardness of both tested materials decreased over time [94].

In addition, Mushashe et al. [95] showed that its impact on the restorative materials was related to the material parameters, and all the changes in the properties of the materials were caused by biodegradation. Therefore, it is certain that although each material mounts different responses to microorganisms, the oral environment can impact the surface characteristics of restorative materials.

## Intelligent dental material surfaces: current situation and prospects

According to previous research, intelligent surfaces that can reduce bacterial adhesion could be designed nowadays. Controlling temperature and magnetic and electric fields is the common method in this respect.

### Temperature-responsive coatings

The temperature-responsive polymers are materials that can undergo such phase transition. They exhibit a lower critical solubility temperature (LCST). They are dissolved in water when the temperature is below LCST. Once the temperature exceeds the LCST, they become insoluble in water and undergo a phase transition to a collapsed structure. Taking advantage of this feature of temperature-responsive polymers, they are used for surface decoration, endowing the surface with a smart antifouling function.

Cunliffe et al. [96] created a temperature-mediated hydrophilic‒hydrophobic switching surface by grafting three carboxyl-terminated polymers (P1–3) onto the amine-functionalized glass substrates. They demonstrated surface hydrophobicity changes with the phase transitions of thermoresponsive polymers and bacterial (*Salmonella typhimurium* & *Bacillus cereus*) detachment from the surface at temperatures below the LCST. However, in particular, bacterial colonization was found at temperatures below the LCST; however, when the temperature increased, bacterial detachment occurred [97]. In short, the short-term reversibility of bacterial attachment-detachment was achieved. Moreover, these temperature-responsive coatings do not impact the bacteria but remove them on demand.

### Magneto-responsive coatings

Applying magnetic stimuli can tune the surface free energy by reversibly changing the surface morphology and wettability, providing the surface with smart surface protection.

Cao et al. [98] produced a smart, responsive superhydrophobic surface composed of a dense array by combining spray coating. The surface performance of reversible switching was achieved based on the magnetic field-stiffening effect of the MREMPs. The properties of these surfaces (adhesion force and sliding behaviors) strongly relied on the intensity and the mixing ratio of PDMS, iron particles, used for the surface preparation process.

This magnetic response superhydrophobic surface can promote the transport of liquid droplets and also lay a good foundation for the research and development of intelligent liquid-repellent skin and intelligent microfluid, showing a broad application prospect.

Magnetic fields can be easily generated and controlled. For this reason, all the bacteria attached can be removed as activation can be easily carried out in cycles. In addition, the risk of nearby tissue or material damage is low in applying magnetic stimuli.

### Electro-responsive coatings

Similar to magnetic field applications, an electro-controlled smart antimicrobial surface can be created by applying an electrowetting phenomenon, using the piezo effect to switch the surface geometry, changing the conformation of polymer brushes.

Deˇkanovsky et al. [99] invented a switchable antimicrobial dual-action functional material with polypyrrole. When the material was immersed in water, an air gap was formed, which prevented the formation of biofouling. The surface properties switched to a highly hydrophilic state by applying an external electric field, releasing the loaded drug. When the electric field was switched off, and the sample was dried, the intrinsic superhydrophobic state of the material surface was regained so that the bacteria could be easily removed. In addition, the smart antimicrobial material exhibited high flexibility and stretchability; therefore, it is a proper candidate for various medical applications.

Guselnikova et al. [100] reported a smart surface with water/oil adhesion. In the process of experimental research, to better meet the requirements related to the system performance, hydrophobic functional groups are grafted onto the fiber, which can significantly increase the corresponding interface contact angle. At the same time, the adhesion also changes so that the purpose related to performance adjustment can be achieved. This way, the surface wettability could be tuned, changing the surface performance to highly adhesive. Furthermore, with the application of an electric field trigger, fast and reversible switching and adhesion were achieved. Moreover, the created fiber-based coating surface exhibited satisfactory stability against mechanical treatment.

Zeng et al. [101] reported electrically switchable friction. Poly-AHPS, an electro-controllable polymer branched brush, was grafted onto an electrically conductive ITO glass when negatively charged. When positively charged, the two states of poly-AHPS had branched brushes, exhibiting two different friction factors and a medium controllable character.

With the technologies mentioned above, smart activation of surface self-cleaning and bacterial repulsion functionality could be achieved by introducing the superhydrophobicity or water repellence of the material surface on demand. Therefore, the pressing issue is applying these technologies to dental materials. The key work in the future is to explore how temperature-responsive polymer, magneto-responsive polymer, or electro-responsive polymer can be effectively coated onto dental materials. Subsequently, for the technology of temperature-responsive coatings, temperature-responsive polymers, which could exhibit the bacterial detachment status (commonly below the LCST) at the temperature of the oral environment, could be applied to dental materials. This way, the dental material’s self-cleaning functionality was achieved, although the temperature would change due to eating and drinking. For technologies of magneto-responsive coatings or electro-responsive coatings, subsequent use of the external magnetic/electric field triggering could provide an opportunity to create and switch the dental material surface between water-repellent/water-adhesive states and achieve an externally controlled self-cleaning functionality in the oral cavity.

## Conclusions

The formation of dental biofilm is a biologically regulated development process. In addition to biological culture medium and nutrient source, other changes in microbial growth conditions will also affect the structure of biofilm. Moreover, the organisms that make up the biofilm significantly impact the biofilm structure [18]. Therefore, the mechanism needs further exploration [4].

Previous studies have significantly improved the recognition of the materials’ physical, chemical, and mechanical interactions. Apart from the research on the surface charge, hydrophobicity, hydrophilicity, and chemical properties of dental materials, insufficient research is available on how surface stiffness and surface morphology affect bacterial adhesion and biofilm formation. In addition, how these surface performances can impact pellicle formation [102] and how the pellicle changes the surface performance of various dental materials deserve profound studies. Moreover, it is necessary to clarify how the relationship between the surface performance of dental materials and the formation of acquired pellicles affect the strength and mechanical stability of bacterial adhesion to surfaces. Understanding the influence of bacterial adhesion on the local microenvironment and analyzing its mechanism can provide support and a basis for the adsorption regulation of biofilm. For example, biofilm is produced on dentures, which will cause inflammation. Under certain stimulation, mucosal surfaces and resin composites can change the composition of proteins and the flow of the crevicular fluid, altering the composition of microbial communities and promoting specific bacterial adhesion patterns [10]. A better understanding of bacteria, monitoring local surface properties, and adjusting their physiological and adhesive properties to environmental changes is the key to designing new antifouling and antibacterial dental material surfaces.

In the oral cavity, the biofilm containing the host and bacterial proteins apparently influences bacterial adhesion. Biofilm formation is a complex process, and factors such as dietary intake and oral microbial composition significantly affect it. Studying the association between the surface performance of materials and bacterial adhesion has shown that negatively charged, superhydrophobic, superhydrophilic, and nano surfaces can all reduce bacterial adhesion. In addition, some positively charged surfaces can achieve antibacterial performance through coating with antibacterial materials. The research and developments concerning new dental materials are more reliable in the simulated oral environment. Therefore, we should combine different models to simulate the complex oral environment to develop and evaluate novel dental materials. This research field has laid a solid foundation for a profound awareness of bacterial systemic sensing surfaces and is very critical for the research and development of intelligent biomimetic dental materials to reduce biological contamination.
